# Asymmetric Synthesis of Axially Chiral Molecules via Organocatalytic Cycloaddition and Cyclization Reactions

**DOI:** 10.3390/molecules28114306

**Published:** 2023-05-24

**Authors:** Wei-Yun Cai, Qian-Ni Ding, Ling Zhou, Jie Chen

**Affiliations:** Key Laboratory of Synthetic and Natural Functional Molecule of the Ministry of Education, College of Chemistry & Materials Science, Northwest University, Xi’an 710127, China; 202220945@stumail.nwu.edu.cn (W.-Y.C.); m15771729382@163.com (Q.-N.D.)

**Keywords:** organocatalytic, cycloaddition and cyclization, atropisomer construction, axial chirality

## Abstract

Atropisomeric molecules are present in many natural products, biologically active compounds, chiral ligands and catalysts. Many elegant methodologies have been developed to access axially chiral molecules. Among them, organocatalytic cycloaddition and cyclization have attracted much attention because they have been widely used in the asymmetric synthesis of biaryl/heterobiaryls atropisomers via construction of carbo- and hetero-cycles. This strategy has undoubtedly become and will continue to be a hot topic in the field of asymmetric synthesis and catalysis. This review aims to highlight the recent advancements in this field of atropisomer synthesis by using different organocatalysts in cycloaddition and cyclization strategies. The construction of each atropisomer, its possible mechanism, the role of catalysts, and its potential applications are illustrated.

## 1. Introduction

**Scheme 1:**
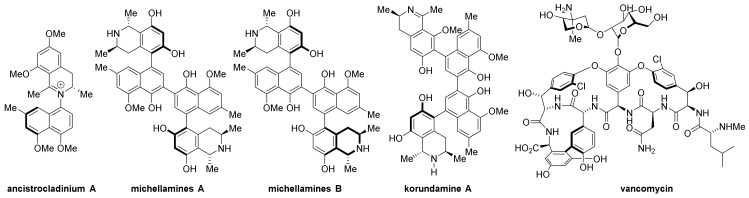
Axially chiral natural products.

**Scheme 2:**
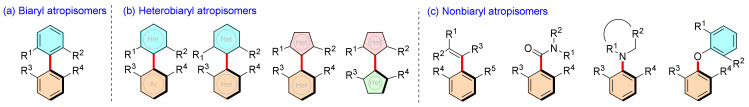
Different type of atropisomers.

Atropisomers are common in natural products and some drugs (Scheme 1) [1,2,3], such as ancistrocladinium A [4], michellamines A and B [5], korundamine A [6], vancomycin, etc. [7]. When two specific groups connected by a σ bond cannot rotate freely due to electronic effects or steric hindrance between them, a pair of immobilized isomers can be separated. The earliest atropisomer was isolated and determined by Christie and Kenner [8], but the definition was not fully developed until Oki’s work [9]. Nowadays, the atropisomers are generally divided into three or four classes according to the type of bond axis (Scheme 2) [10,11].

Biaryl is a structure consisting of two full-carbon aromatic rings connected by a single bond. The chiral ligand 2,2’-bis(diphenylphosphaneyl)-1,1’-binaphthalene (BINAP) with this framework was first applied to the asymmetric hydrogenation performed by Noyori et al. [12]. This application opened the door to the study of biaryls as ligands and catalysts. Since then, binaphthol (BINOL) and other biaromatic ligands have been synthesized and used as privileged chiral ligands in the field of asymmetric metal catalysis. Then, in 2004, Akiyama [13] and Terada [14] independently developed BINOL-derived chiral phosphoric acid catalysts and successfully catalyzed the asymmetric Mannich reaction with these catalysts, which is an important breakthrough for the application of biaryls in the field of organocatalysis.

Heterobiaryl has one or more heteroatoms on the aromatic ring, which allows it to participate in reactions through the action of heteroatoms and other molecules without dependence on substituents. This property often makes this skeleton catalyst uniquely able to catalyze some reactions. In 1991, the first heterobiaromatic N,P-ligand (1-(1-isoquinolinyl)-2-naphthyl)diphenylphosphine (QUINAP) was successfully synthesized [15], followed by a series of other ligands based on this skeleton, such as 1-(isoquinolin-1-yl)naphthalen-2-amine (IAN), and some heterobiaryls with good organocatalytic properties were synthesized [16,17], which greatly expanded the choice of catalysts [18].

In addition to traditional benzamides and styrenes, nonbiaryl anilide derivatives containing C-N axial chirality have been developed over the past two decades [19]. These structures exist widely in natural products and have wide application in medicine [20]. Diaryl ether, another type of nonbiaryls containing C-O-C bonds, was proved by Mislow et al. in 1982 [21], but its synthesis was not reported until 1998 by Kinoshita et al. [22]. At present, there is still a large room for research on this kind of compounds, and new progresses have been developed for the synthesis of these compounds by using organocatalytic strategies in recent years [23].

Due to its unique steric hindrance, atropisomer has an irreplaceable role in metal catalysis and organocatalysis [24]. As a result, its asymmetric synthesis has become a hot topic in the field of organic synthesis [25]. The existing synthetic methods mainly include coupling, aromatic ring construction, chiral separation/desymmetrization and chiral transformation [26,27,28,29,30]. Cycloaddition and cyclization are common methods in the ring construction, which are efficient and environmentally friendly synthetic methods [31,32]. Organocatalytic cycloaddition and cyclization adhere to this concept, using efficient and environmentally friendly small molecular organocatalysts to achieve this goal.

Organocatalysis is no stranger to chemists; its mild conditions, low expense and easy to obtain, environmental-friendliness, excellent atomic economy make it an ideal and feasible catalytic strategy, as well as in the construction of atropisomers. Most of these reactions are catalyzed by Brønsted acid or Lewis base [33], which can form intermolecular hydrogen bonds, facilitate the reaction by giving or receiving protons, and control the reaction direction of substrate through steric hindrance of groups or an inductive effect of hydrogen bonds, so as to obtain single configuration of atropisomers. In the process of organocatalytic cycloaddition/cyclization, it is usually necessary to go through multiple intermediates and involve the formation of multiple bonds, so the requirements for organocatalysts are more stringent. Under the joint efforts of various research groups, many kinds of organocatalysts have been developed and applied in synthetic chemistry [34,35]. Based on the types of organocatalysts used to achieve cycloaddition and cyclization reactions for the synthesis of axially chiral molecules, this review focuses on the activation mode of catalysts and substrates and the intermediates formed in some representative reactions, and the application of some products is then briefly introduced.

## 2. Chiral Phosphoric Acids (CPAs)

**Scheme 3:**
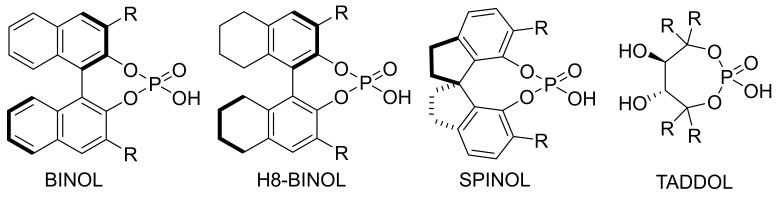
Classification of CPAs.

The chiral part of chiral phosphoric acid mainly consists of BINOL, H_8_-BINOL, SPINOL and TADDOL (Scheme 3) [36,37]. Different catalysts can be derived from different substituents on aromatic rings. Each CPA catalyst has a slight difference in steric hindrance and inductive effect [38]. The functional part of the catalyst contains both hydroxyl sites as proton donors and oxygen sites as proton acceptors [39]. These enable CPA catalyst to adapt to a variety of reactions, and to change its skeleton and substituents according to the needs of different reactions, so that it can be better combined with the substrates and catalyze the reaction.

**Scheme 4:**
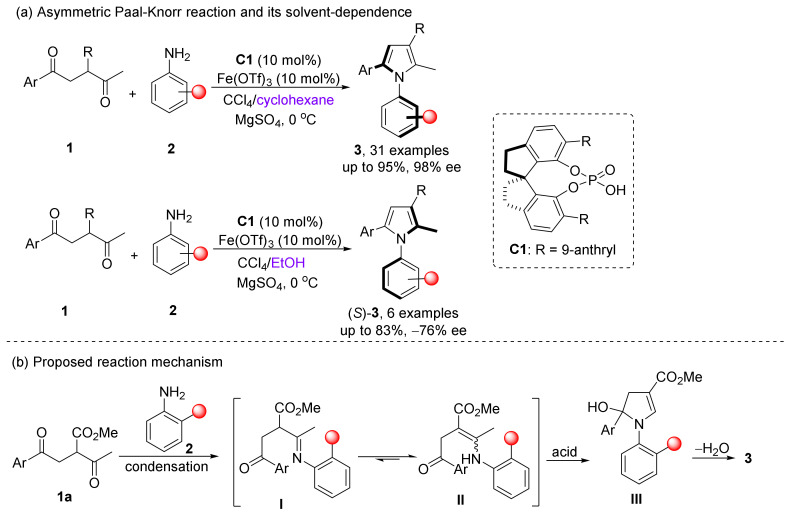
Asymmetric Paal–Knorr reaction to synthesize N-arylpyrroles.

Tan et al. made an early start in using CPAs to catalyze asymmetric cycloaddition/cyclization to construct axially chiral molecules. In 2017, they reported an asymmetric Paal–Knorr reaction to synthesize arylpyrroles, giving up to 95% yield and 98% ee (Scheme 4) [40]. This general and efficient reaction has mild condition and wide adaption to many substituent groups. Remarkably, during screening solvents, they found that the configuration of the product depended on the solvent, wherein cyclohexane and ethanol gave opposite results. After further optimization, the product of *S*-configuration could be stably obtained, although its yield and enantioselectivity were relatively low. Moreover, both of the reaction efficiency and enantioselectivity were promoted when Fe(OTf)_3_ was used as an additive, wherein the phosphoric acid **C1** was activated by the Lewis acid. Further investigation shows **1a** is first condensed with **2**, and after undergoing the equilibrium of intermediates, enamine **II** is generated, and then the product is obtained by the second condensation cyclization. This process is different from the Paal–Knorr reaction mechanism previously thought.

**Scheme 5:**
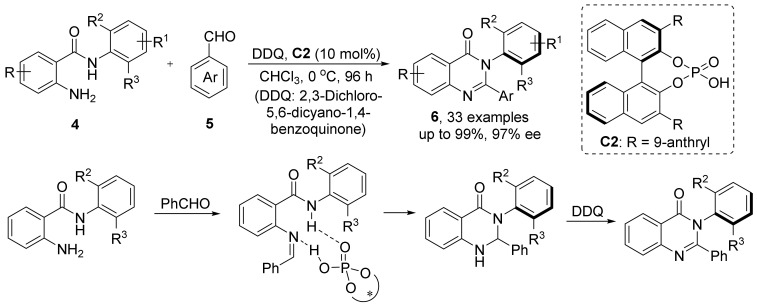
Tan’s arylquinazolinone synthesis and its reaction mechanism.

In the same year, Tan et al. reported a cyclization strategy catalyzed by CPA for the construction of arylquinazolinones (Scheme 5) [41]. They successfully synthesized arylquinazolinones with excellent yields and enantioselectivities using N-aryl anthranilamides and benzaldehyde as substrates under CPA catalysis. In the reaction process, N-aryl anthranilamides were first condensed with benzaldehyde, and the condensation products formed hydrogen bonds with CPA. Under the induction of CPA, amido N atom conducted nucleophilic attack on imine C atom, and a proton exchange occurred with CPA to complete the ring closure.

**Scheme 6:**
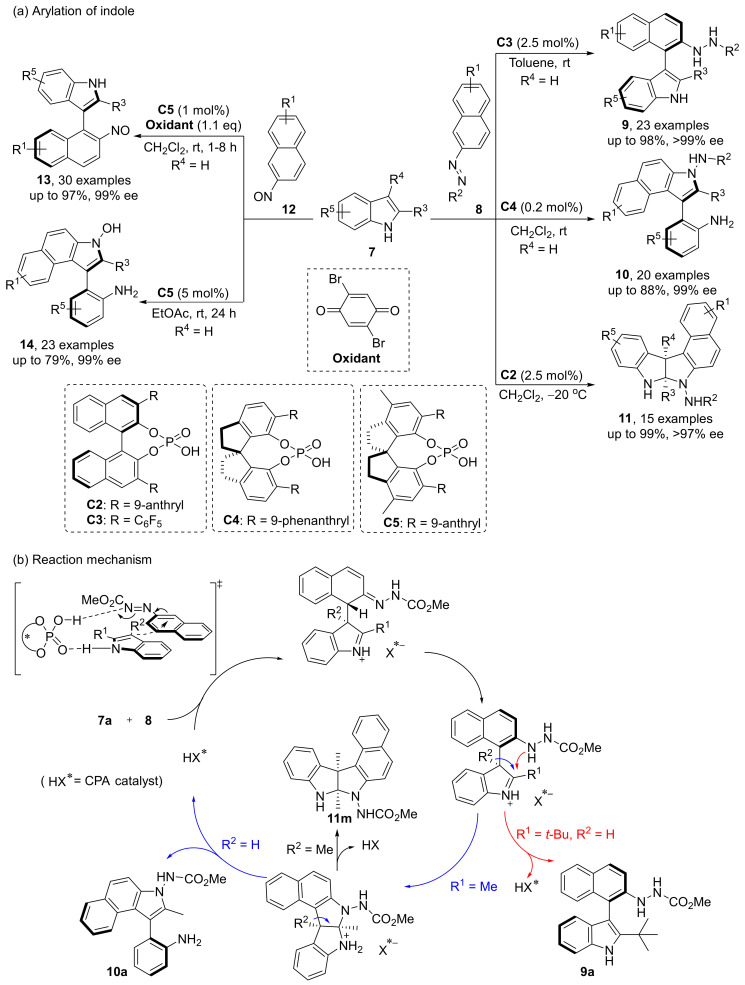
Nucleophilic reaction of indoles.

They also developed a nucleophilic aromatic substitution method for the synthesis of arylindoles in 2018 (Scheme 6) [42]. In this process, they accidentally discovered that under certain conditions, coupled intermediates could generate new axially chiral aniline indoles by ring opening and cyclization. This reaction has high reaction rate, mild conditions, low catalyst loading, good yield and enantioselectivity. In order to expand the scope and application of this reaction, they selected new nitroso aromatic substrates with the help of DFT guidance in 2020, and successfully obtained corresponding products with excellent yields and enantioselectivities [43]. Mechanistic studies showed that the nucleophilic attack of indole on naphthalene C1 position catalyzed by CPA leads to the dearomatization of indole and naphthalene ring. Then, depending on the steric hindrance of substituted R^1^ group, the nucleophilic attack on indole ring by N atom or the direct deprotonation to give product **9** may occur. The intermediate obtained through the nucleophilic attack will directly generate the centrally chiral product **11** or transform into the axially chiral product **10** according to the different R^2^ groups.

**Scheme 7:**
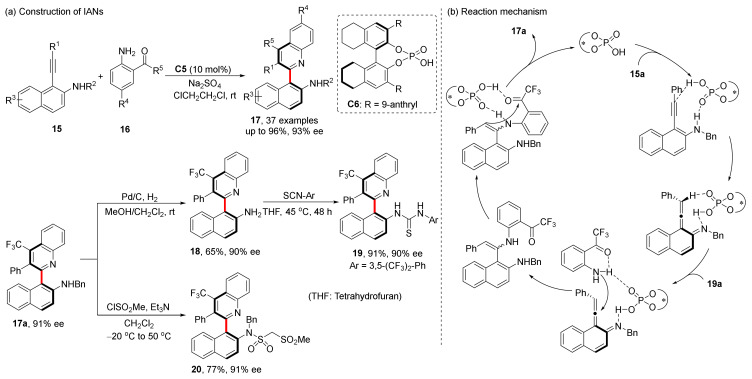
Synthesis of IANs.

In 2020, they designed a construction of IAN analogues through vinylidene *ortho*-quinone methide (VQM)-like intermediates (Scheme 7) [44]. CPA acted as both Brønsted acid and base through the formation of hydrogen bonds with the substrates. In this reaction, phosphoric acid provided proton transfer and chiral induction in the formation of VQM-like intermediates. The reaction is compatible with up to 37 substrates with excellent yields and enantioselectivities, and the resulting atropisomeric C2-arylquinoline skeletons can be converted into multiple functional axially chiral molecules while preserving good ee values. Notably, CPA-catalyzed heteroannulation of alkynyl naphthanols and aminobenzaldehydes gave only racemic heterobiaryls due to a lower rotation barrier of OH group [45].

**Scheme 8:**
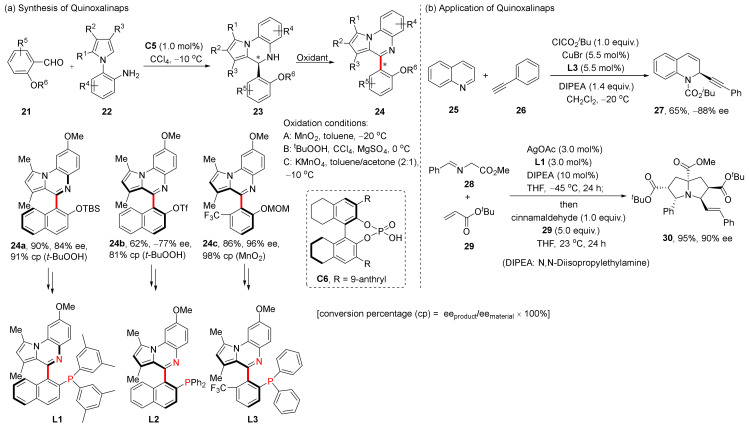
Synthesis and application of Quinoxalinaps.

In terms of the construction and application of axially chiral quinoline derivatives, Jiang et al. tend to make them into new N,P ligands. In 2021, based on previous work using CPA catalyzed dynamic kinetic resolution (DKR) to construct multiaxially chiral compounds [46], they reported a new class of N,P-ligands Quinoxalinaps, and successfully constructed a template reaction for the synthesis of such ligands by using the central-to-axial chirality conversion strategy (Scheme 8) [47]. Through this strategy, CPA catalyzed cyclization of **21** and **22** gave enantioenriched compounds **23**; then, oxidation dehydrogenation using different oxidants yielded axially chiral products **24**. In subsequent studies, they used this method to construct a variety of Quinoxalinap ligands; all of them showed excellent performance as transition metal catalytic ligands, which could catalyze a variety of reactions efficiently. In the same year, they reported a new family of axially chiral heterobiaryls and the N,P-ligand quinazolinonaps derived from them [48]. Three different methods involving central-to-axial chirality conversion, palladium-catalyzed DKR, and asymmetric phase-transfer catalysis were used to synthesize these ligands.

**Scheme 9:**
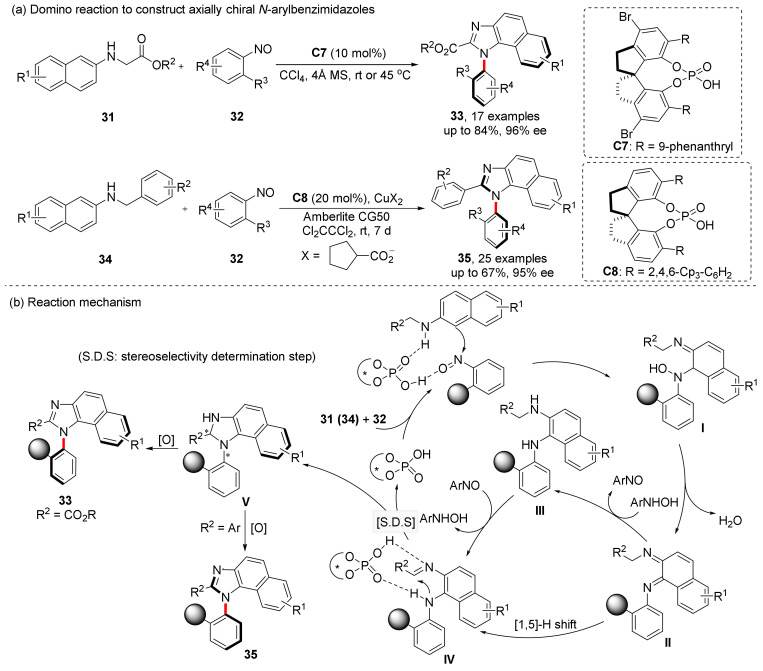
Domino reaction to synthesize axially chiral imidazoles in one step.

Because of the lower configurational stability, the construction of atropisomers bearing five-membered heterocycles has been a challenge to researchers. Tan et al. reported the construction of axially chiral imidazole compounds using domino reaction of 2-naphthylamine derivatives and nitrosobenzene derivatives in 2021 (Scheme 9) [49]. This strategy has simple steps and mild reaction conditions. The reaction can be completed in an hour, and the enantioselectivity is up to 97% ee, and 95% ee can also be achieved when substrate **37** was used. The key to this strategy is the use of nitroso group. As a nucleophilic and electrophilic reagent, nitroso is attacked by the nucleophilie of the naphthalene ring, and then acts as a nucleophile to attack the C atom of the imine to complete the ring closure. Nitroso also acts as an oxidant, facilitates the oxidative aromatization of the last step of the reaction. Depending on the R^2^ substituents, the products will have different configurations, and this result may originate from the different non-covalent interactions (H-bond interaction for ester group and π–π stacking interaction for aryl group).

**Scheme 10:**
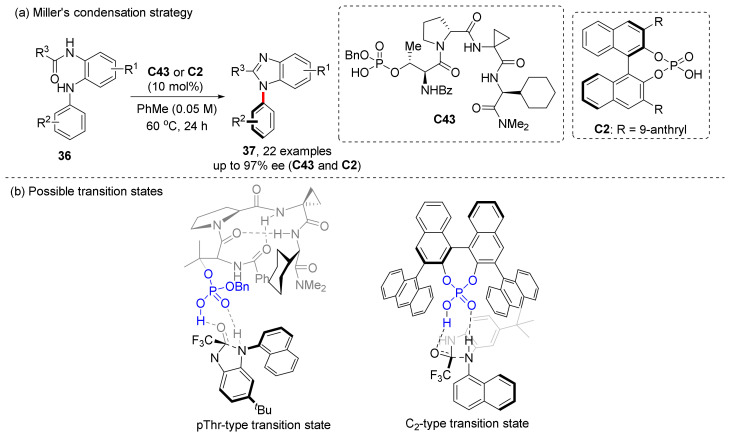
Miller’s condensation strategy for axial chirality synthesis.

In terms of the construction of the imidazole ring, Miller et al. had already reported a successful case in 2018 [50]. In 2019, they further compared the two catalysts used for condensation from both experiments and calculations, and found that their catalytic effects were similar, but slightly different in mechanism (Scheme 10) [51]. Biaryl C2-type CPA catalysts determine enantioselectivity through steric effects of large groups, while pThr-type CPA catalysts work through an alternative mode of enantioinduction.

**Scheme 11:**
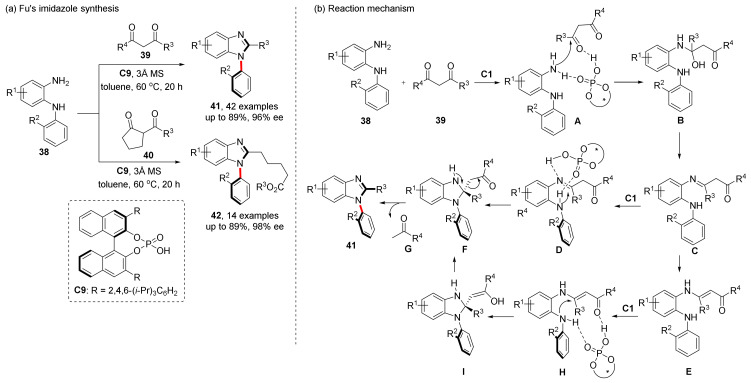
C-C bond cleavage strategy to synthesize N-arylbenzimidazoles.

In 2020, Fu et al. also challenged the synthesis of chiral imidazoles (Scheme 11) [52]. Different from the strategy of both Miller and Tan, they used CPA to selectively cleave carbon–carbon bonds. A series of *N*-arylbenzimidazoles were successfully synthesized with up to 96% ee by using acetylacetone as a substrate due to its high activity of multicarbonyl compounds. In this reaction, the carbonyl group on one side of multicarbonyl compounds **39** firstly forms hydrogen bond with the CPA, and condensing with diamines **38** to give imine **C**. Then, intramolecular addition to the imino group affords **F**. Alternatively, isomerization of **C** gives enamine **E**, and then the Micheal addition occurs to also give intermediate **F**. Finally, the C-C bond cleavage of **F** to remove **G** and finish aromatization to obtain product **41**.

**Scheme 12:**
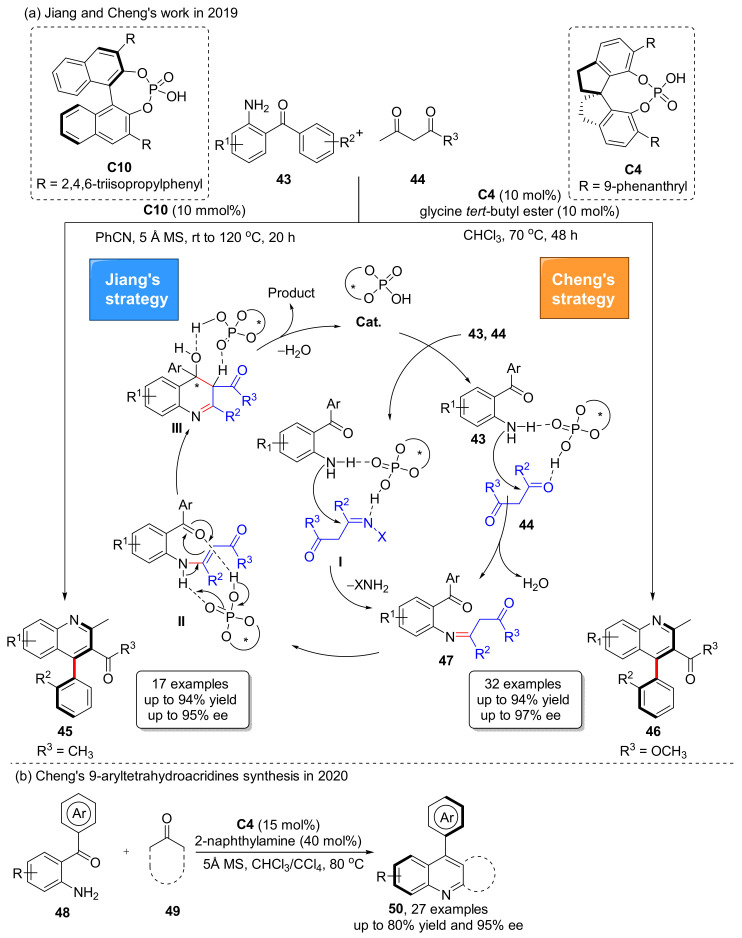
Asymmetric Friedlander hetero-annulation reaction to access 4-arylquinolines.

In 2019, both Cheng et al. and Jiang et al. reported their discovery in atroposelective Friedlander quinoline heteroannulation to construct 4-arylquinoline architectures, and it exhibited a wide range of substrates. A wide variety of 4-arylquinolines were prepared with excellent enantioselectivities (Scheme 12a) [53,54]. The focus of asymmetric synthesis is the chiral induction cyclization to form intermediate **III**. Catalyzed by CPA, the product is aromatized by removing one molecule of water, which is also a key step in the central-to-axial chirality conversion. The following year, in 2020, Cheng et al. successfully extended Friedlander reaction by using a similar method to synthesize 9-aryltetrahydroacridines **50** (Scheme 12b) [55]. They successfully obtained up to 80% yield and 95% ee with the addition of 2-naphthylamine using the same catalyst.

**Scheme 13:**
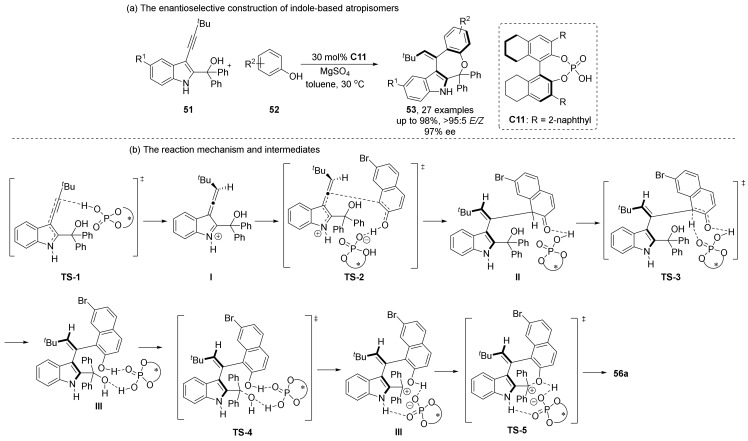
CPA-catalyzed enantioselective [4+3] cyclization of aryl-acetylenyl indoles.

Shi et al. also conducted a lot of research on the asymmetric cycloaddition/cyclization to construct axially chiral molecules catalyzed by CPA. In 2020, they reported an axially chiral aryl-alkene-indole framework through an allene-iminium intermediate (Scheme 13) [56]. The framework is synthesized via (*Z*/*E*)-selective and enantioselective (4 + 3) cyclization of aryl-acetylenyl indoles **51** with phenols **52** catalyzed by H_8_-BINOL-type CPA. CPA as Brønsted acid catalyzed substrates’ conversion to allene-iminium intermediates **I**. In the transition state **TS-2**, substrate **52** attacks from the H side with low steric resistance; thus, the *Z*/*E* configuration of the olefin is determined. Finally, under the catalysis of CPA, two hydroxyl groups of intermediate **III** are condensed into ethers to complete the reaction.

**Scheme 14:**
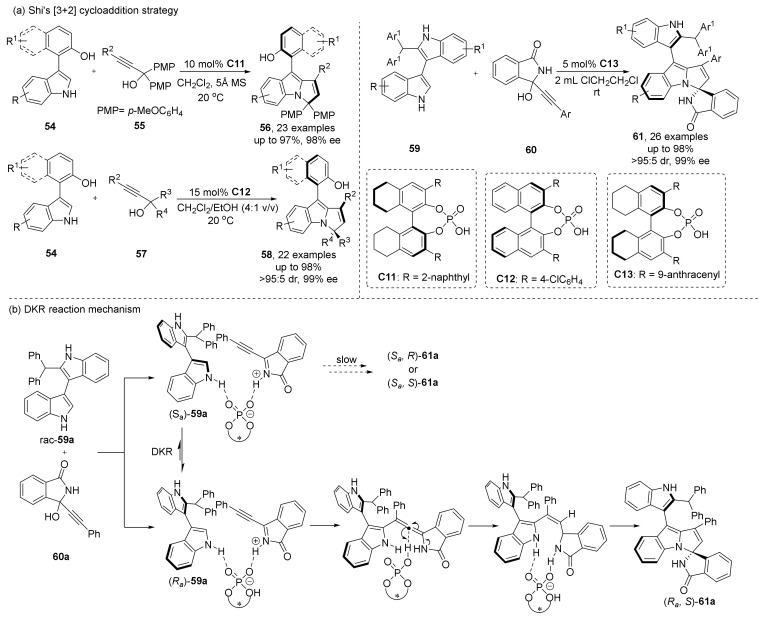
CPA-catalyzed [3+2] cycloaddition to construct central and axial chirality.

The following year, they designed a [3+2] cycloaddition reaction to synthesize 3-arylindoles **56** and **58** with axial chirality by means of DKR (Scheme 14) [57], in which an R^2^ group with higher steric hindrance was used, resulting in the indole-aryl bond rotation obstruction, thus achieving the purpose of constructing axial chirality. Under the catalysis of CPA, allene-type intermediates are formed from *p*-QM intermediates. When the PMP is replaced by different groups, the addition to allene intermediates also produces central chirality, and its diastereoselectivity can be controlled around 10:1 dr. They also synthesized indolyl-pyrroloindoles **61** with central and axial chirality at the highest 95% yield, 91:9 dr and 99% ee [58].

**Scheme 15:**
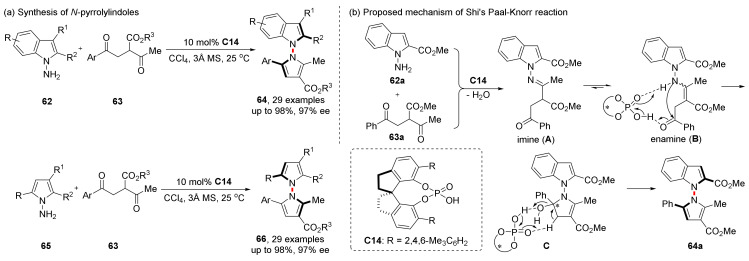
Paal−Knorr reaction to construct axially chiral aryl-pyrroloindoles.

Very recently, they have also joined in the construction of N-N axial chirality and achieved a series of results. In 2022, they successfully designed and synthesized the axially chiral aryl-pyrroloindoles **64** by constructing pyrrole rings based on *N*-aminoindole using CPA catalyzed asymmetric Paal−Knorr reaction (Scheme 15) [59]. Interestingly, the optimal catalyst they selected and the catalyst Tan et al. adopted in 2017 both belonged to SPINOL type CPA; however, only the R group on the aromatic ring changed. The reaction is also suitable for bispyrrole synthesis (**66**), which can be generated in 98% yield and 97% ee under the same conditions. In addition, they proposed a more detailed reaction mechanism, in which the catalytic activation of CPA as Brønsted acid can be intuitively seen. The formation process of the chiral axis of pyrroloindoles can also be clearly understood.

**Scheme 16:**
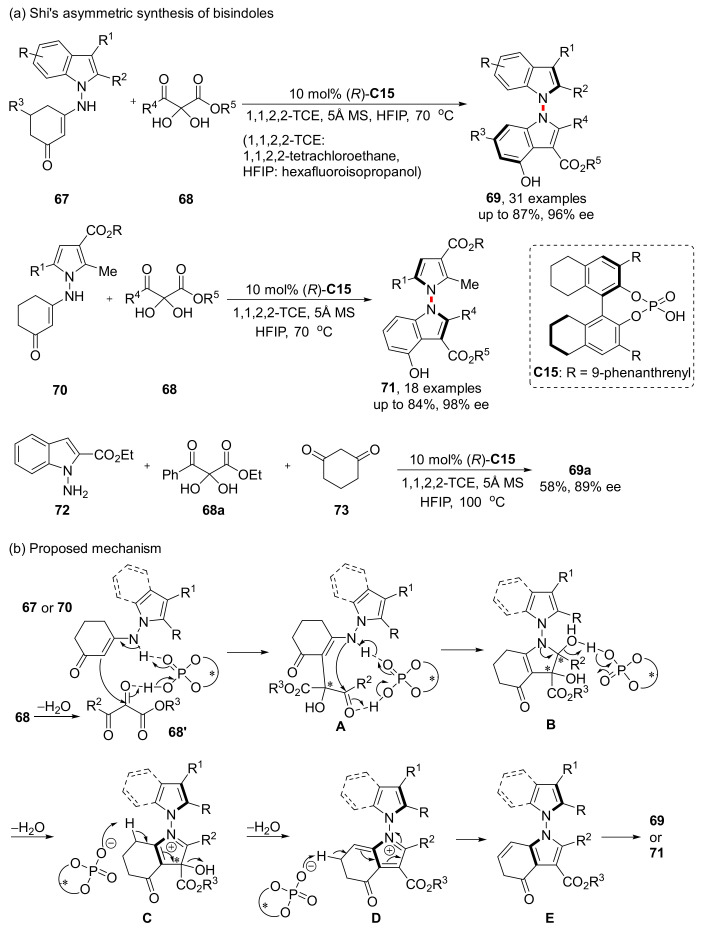
Synthesis of axially chiral *N,N’*-bisindoles.

On the basis of the results mentioned above, the asymmetric synthesis of axially chiral *N,N’*-bisindoles was designed and completed in 2023 (Scheme 16) [60]. Reactions can be performed using pyrrole-based enaminone **70** with 2,3-diketoester precursors **68**, with a slightly better enantioselectivity but a slightly lower yield than indole-based substrate **67**. They also tried a one-pot synthesis of bisindoles from ingredient **68a**, **72** and **73**, and the results were apparently successful, with the reaction proceeding as scheduled in 89% ee. It can be seen from the proposed mechanism that CPA is involved in every transformation and plays an important role in this process, but only the formation of the two central chirality in the intermediate chiral parts is actually involved.

**Scheme 17:**
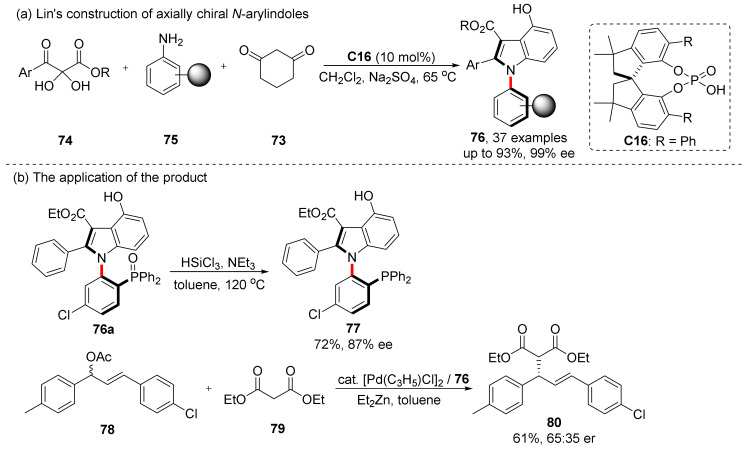
Lin’s construction strategy with their characteristic CPA catalyst.

The construction of chiral *N*-arylindoles via CPA catalyzed cyclization has been proven to be an efficient approach. Lin et al. had reported similar reactions as early as 2019 (Scheme 17) [61]. They performed a CPA catalyzed three-component reactions using 2,3-diketoesters **74**, aryl amines **75** and 1,3-cyclohexanedione **73**. Their strategy has excellent yields and enantioselectivities. This reaction was compatible with a variety of functional groups, and the product **76a** could be converted into a chiral phosphine ligand and had a good catalytic effect on the Pd catalyzed allylation of diethyl malonate with **78**. It is worth mentioning that the CPA catalyst they used was a new TM-SPINOL type catalyst independently developed by themselves, and this reaction proved that this class of catalysts they developed had high selectivity in similar asymmetric reactions.

**Scheme 18:**
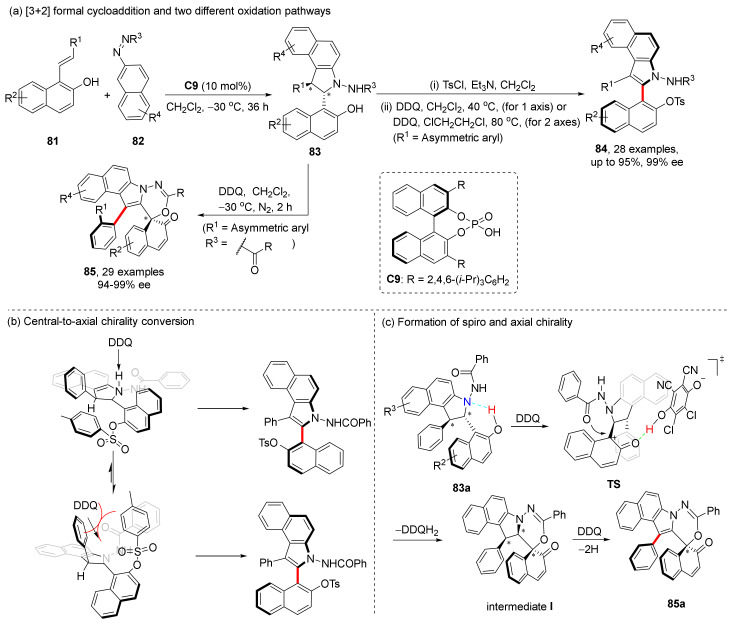
[3+2] cycloaddition and oxidation to synthesize heterobiaryls with two chiral elements.

Our research group has long been committed to constructing axially chiral compounds using asymmetric cycloaddition/cyclization strategies catalyzed by CPA. In 2019, we reported a chiral phosphoric acid-catalyzed [3+2] formal cycloaddition and a mild DDQ oxidation strategy (Scheme 18a) [62]. The core of this strategy lies in the oxidation of two chiral centers into one or even two chiral axes through the central-to-axial chirality conversion. We found that the configuration of the final product was different with the stable conformation of the central chiral cycloaddition product, which was confirmed via NMR experiments and DFT calculations (Scheme 18b). Furthermore, DFT calculations indicate that the hydride transfer is the rate-determining step of the overall transformation.

Further, in 2021, we developed an oxidation strategy to realize both central-to-axial chirality conversion and central-to-spiro chirality transfer under the oxidation of DDQ [63]. In which oxidation of unprotected naphthanols **83** gave products **85** with excellent enantioselectivities. Based on control experiments and DFT calculations, a reasonable mechanism was proposed (Scheme 18c). DDQ oxidation of a naphthyl ring was employed to form a carbocation intermediate via a dearomatization pathway; then, the spiral chirality was formed by a nucleophilic attack of carbonyl oxygen atom on carbocation. Final oxidation was completed to form the axial chirality. Notably, the hydrogen bonds play important roles in this process.

**Scheme 19:**
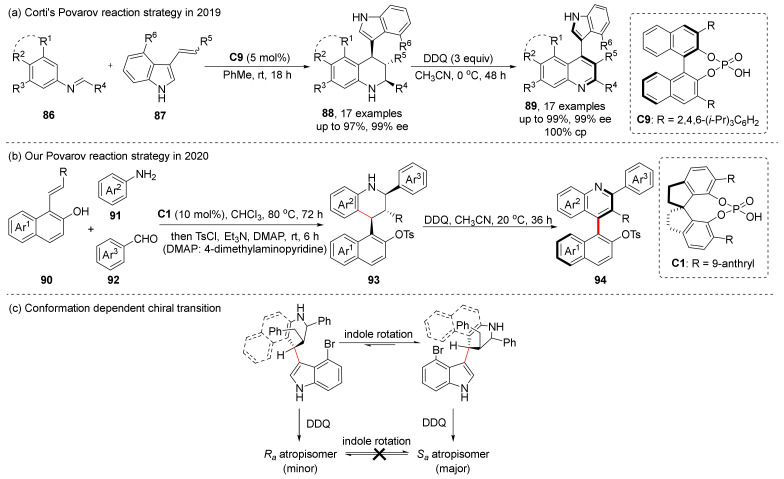
Asymmetric Povarov reaction and its oxidation.

We also tried the CPA-catalyzed asymmetric [4+2] Povarov cycloaddition and central-to-axial chirality conversion in 2020 and achieved good results (Scheme 19b) [64]. In addition, we also attempted to construct multiple chiral axes via oxidation with one step, and the results were as expected, showing high yields, high diastereoselectivities (>20:1 dr) and high enantioselectivities (99% ee), which may be widely used in the synthesis of chiral catalysts in the future.

In 2019, before we reported on this reaction, Corti et al. reported a similar case of synthesis of axially chiral indole-quinolines using Povarov cycloaddition and central-to-axial chirality conversion (Scheme 19a) [65]. In the central-to-axial chirality conversion step, they found that the configuration of the final product was the same as the stable conformation of the central chiral cycloaddition product, which was confirmed via the DFT calculations.

**Scheme 20:**
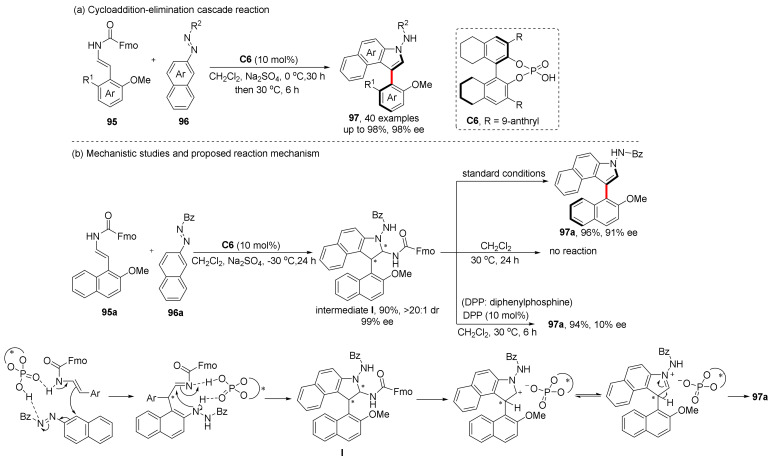
CPA-catalyzed cycloaddition–elimination cascade reaction.

In the construction of axially chiral compounds, the asymmetric cycloaddition strategies using alkenes as substrates usually require a further chirality conversation step from centrally chiral compounds formed after cycloaddition. For example, the above work had to introduce exogenous oxidants. This strategy usually requires oxidants with matching oxidation properties; otherwise, various side reactions are likely to result in low yield or racemization. On the other hand, the removal of small molecules by elimination is another common strategy, which needs ingenious substrate design and requires suitable active substrates and leaving groups. Based on our previous work, we designed a cycloaddition–elimination cascade reaction catalyzed by CPA (Scheme 20) [66]. A carbamate attached to the alkene substrate as the leaving group can be eliminated under the acidic condition provided by CPA. Thus, CPA-catalyzed cycloaddition of **95** with **96** was first achieved. By controlling experimental conditions, the centrally chiral intermediate **I** can be obtained at a low temperature. Subsequent control experiments show that CPA plays an important role in the next elimination step, wherein central-to-axial chirality conversion was completed.

**Scheme 21:**
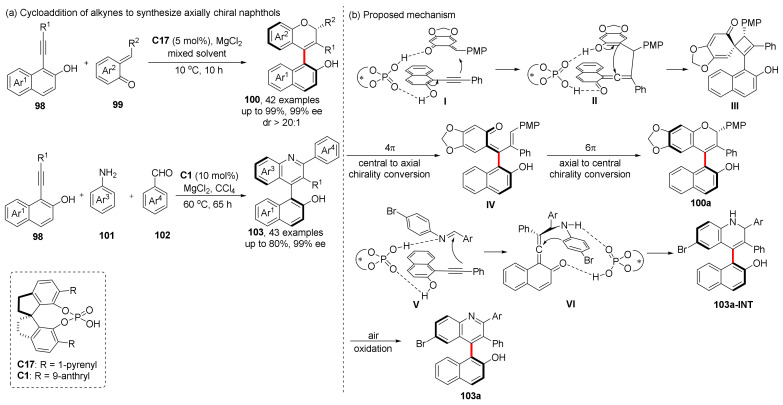
CPA-catalyzed cycloaddition of alkynyl naphthanols.

Alkynes have a higher degree of unsaturation than alkenes, which can be used to construct axially chiral molecules in one step by means of cycloaddition to avoid side reactions and losses during subsequent oxidation or elimination [67]. It is an ideal substrate for the construction of aromatic rings in cycloaddition reactions where atomic economy is high. However, alkynes are often difficult to participate in organocatalytic reactions due to their poor nucleophilic activity, which is a challenge for alkyne addition reactions. In 2022, based on a reactive VQM intermediate reported by Irie et al. and Yan et al. [68,69,70,71], we reported all-carbon tetrasubstituted VQM intermediates formed from *o*-alkynylnaphthols and their conversion applications (Scheme 21) [72]. During the formation of this intermediate, the substrate alkynyl naphthol first attacks the *o*-quinone methides (*o*-QM) to form VQM structure under the dual H-bonding activation of chiral phosphoric acid, and, at the same time, restores aromatization. The intermediates are dearomatized and the intramolecular nucleophilic attack occurs to form a four-membered ring (**III**); then, electrocyclization occurs twice (opening and re-closing) to form the final product **100a**. The reaction mode of aniline is relatively simple, after the condensation of aniline and benzaldehyde, under the activation of double hydrogen bonds of chiral phosphoric acid, the alkyne substrate attacks imine to give VQM intermediate **VI**, and the intermediate is then attacked by the *ortho* C atom of aniline to obtain the intermediate **103a-INT**, which cannot be separated and is quickly oxidized by air into the final product.

**Scheme 22:**
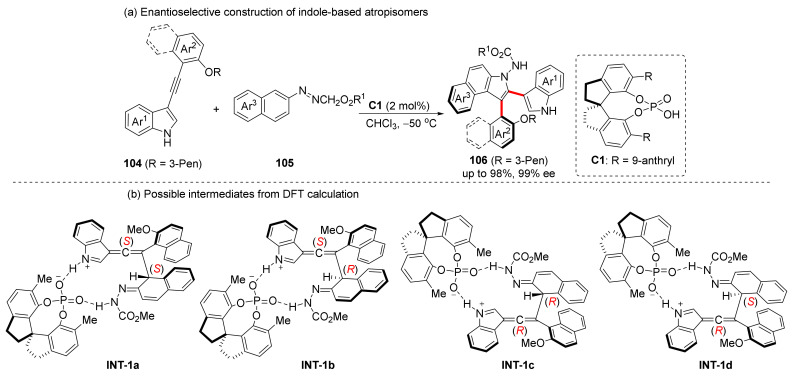
Cycloaddition of alkynyl indoles via allene-iminium intermediates.

For some less electrophilic substrates, naphthol is still insufficient to enable electron transfer of alkynes. The heterocyclic ring of the indole is relatively more electron-rich, allowing the alkynes to react more easily with some less electrophilic substrates through inductive effects, thus extending the range of substrates. Therefore, in 2022, we presented a [3+2] cycloaddition of 3-acetylenyl indoles and azo compounds to synthesize axially chiral indole-aryl compounds through allene-iminium intermediate (Scheme 22a) [73], which is similar to VQM in structure. The products with up to 98% yield and 99% ee can be converted into a variety of ligands and catalysts while maintaining their axial chirality. DFT calculation confirmed the existence of allene intermediates and gave the relative activation-free energy of transition states. It is worth noting that there are two chiral elements in the intermediate according to the DFT-calculation, and these two chiral elements are likely to be retained in the reaction, which means that this synthesis strategy is expected to synthesize biaxial atropisomers in one step. In fact, we have conducted relevant experiments; however, the conditions still need to be optimized.

## 3. Cinchona Alkaloids Derivatives

**Scheme 23:**
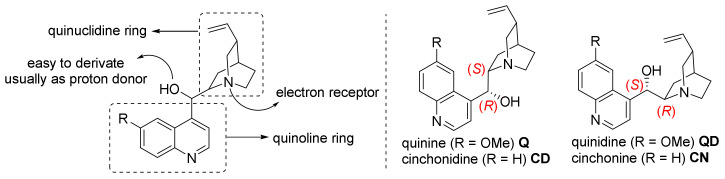
Cinchona alkaloids.

Cinchona bases are a group of natural alkaloids found in the bark of cinchona plants. About 30 cinchona bases are known; however, the following 4 have received the most attention: quinine, quinidine, cinchonine, and cinchonidine (Scheme 23) [74,75]. These compounds are usually divided into quinoline ring and quinuclidine ring. The N atom in the latter serves as proton receptor, and the connecting part in the middle is often converted into a group that can give proton due to its hydroxyl group; therefore, it has a similar catalytic effect to CPA. At the same time, the advantages of high yield and low price also make them a widely used organocatalyst in asymmetric cycloaddition/cyclization reactions.

**Scheme 24:**
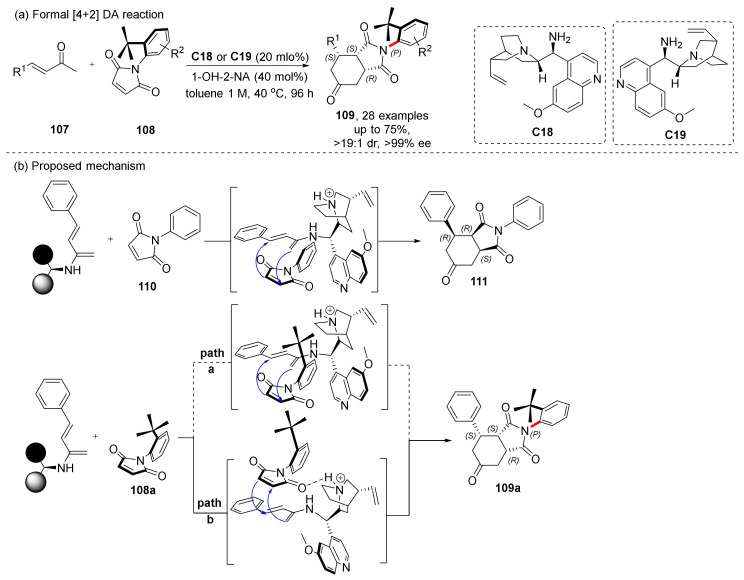
Formal DA cycloaddition desymmetrization.

The earlier use of this catalyst for asymmetric synthesis of axially chiral molecules via cycloaddition/cyclization was reported by Bencivenni et al., who used this type of catalyst to complete the Diels−Alder (DA) desymmetrization of *N*-arylmaleimides in 2015 (Scheme 24) [76]. Based on earlier studies on desymmetrization of *N*-arylmaleimides as substrate [77], they proposed a method through regionally selective desymmetrization results in C-N axially chiral compounds. The chiral amine catalyst derived from cinchona alkaloids is first condensed with the conjugated diene, and the selective synthesis of a single enantiomer is achieved via H-bonding and steric hindrance. They also carried out a significant amount of research on desymmetrization with this type of catalyst, which is only listed for readers to consult as it is not relevant to the topic of this review [78,79].

**Scheme 25:**
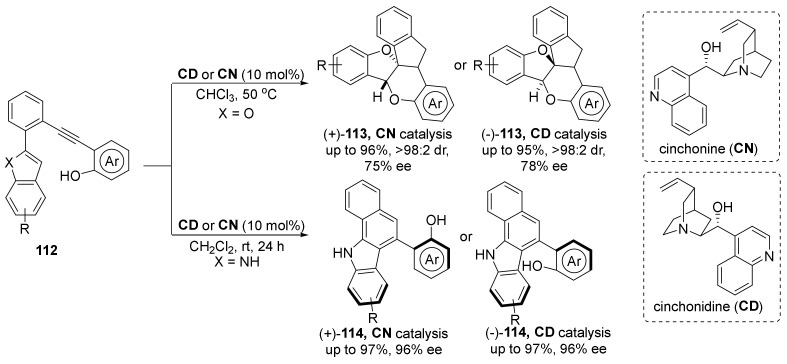
Synthesis of axially chiral benzocarbazoles catalyzed by chiral bases.

The cinchona alkaloids typically start cycloaddition/cyclization reactions as Brønsted bases, and the hydroxyl group forms a hydrogen bond with the substrate, which, together with the quinuclidine ring, controls the chirality of the product. In 2018, based on their previous work, Irie et al. reported a transition-metal-free approach to axially chiral benzocarbazole derivatives (Scheme 25) [80,81]. Different from their work in last year, they changed the benzofuran into the indole (**112**), which increased the yield of axially chiral product. It is speculated that the benzofuran is easier to be dearomatized than indole, due to indole’s larger aromatic stabilization energy.

**Scheme 26:**
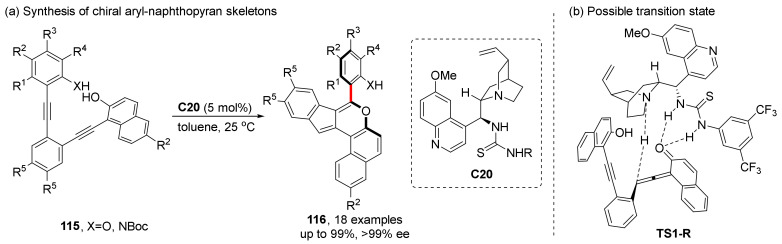
Intramolecular cycloaddition of 2-ethynyl-phenols.

When it comes to the asymmetric cycloaddition/cyclization to construct axial chirality catalyzed by cinchona alkaloid derivatives, Yan et al.’s work in related aspects is very representative. In 2018, they achieved atroposelective synthesis of axially chiral aryl-naphthopyran skeletons through intramolecular [4+2] cycloaddition of 2-ethynyl-phenols (Scheme 26) [82]. Under the activation of alkaloid-derived thiourea catalyst **C20**, products with excellent yields and enantioselectivities (99% yield and 99% ee) are obtained, and the reaction adapt to up to 18 substrates, scale-up experiment gives 98% yield and over 99% ee, which confirms its application potential in industry. Proposed transition state and intermediate show that alkaloid part and thiourea part individually act as Brønsted base and Brønsted acid, and the configuration is controlled by H-bonding between the catalyst and the substrate.

**Scheme 27:**
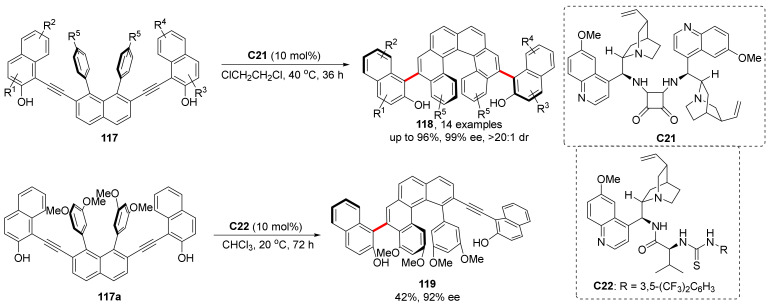
Helical and axial chirality synthesis through VQM intermediates.

Inspired by Irie’s pioneering work and based on their previous work, they reported a novel strategy to construct molecules (**118**, **119**) with both helical and axial chirality (Scheme 27) [83]. Mechanistic studies reveal that the reaction can be taken apart into two processes, and both of the processes can be regarded as a simple cyclization reaction through VQM intermediates. In fact, the two axes have same configuration, and the reaction can be kept at the first step by controlling the reaction conditions. After the first cyclization finished, tetrahelicene-like intermediate generates a helix and another steregenic axis under the catalyst control.

**Scheme 28:**
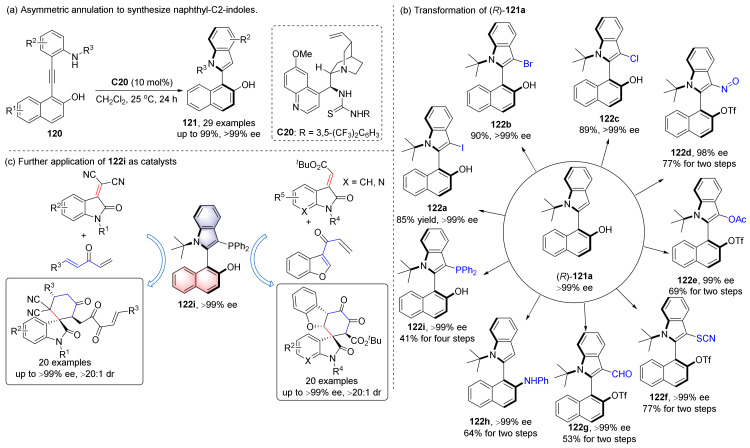
Synthesis of naphthol-C2-indoles and its application.

In 2019, they also explored the enantioselective synthesis of heterobiaryls, and successfully synthesized naphthol-C2-indoles **121**, most of the substitutions of which were at the C3 position of the indole before (Scheme 28) [84,85]. The reaction has excellent enantioselectivities (>99% ee), good performance in decagram-scale reaction, and the yield is higher than 90%. Moreover, the product can be transformed into up to nine types of functional atropisomers while maintaining its chirality. One of them, **122i,** can be used as a chiral phosphine catalyst for aza–Baylis–Hillman reaction and asymmetric formal [4+2] cycloadditions, has broad substrate adaptability, excellent enantioselectivity and diastereoselectivity.

**Scheme 29:**
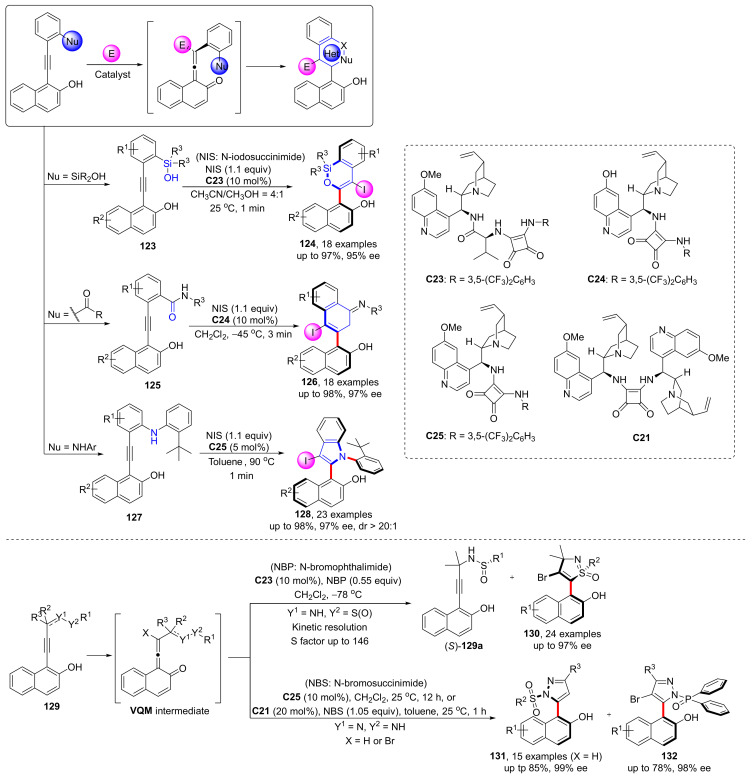
Template reaction of VQMs.

Based on the previous synthesis of chiral heterobiaryls, in 2021 and 2022, they designed a strategy to construct chiral heterobiaryls via construction of a heterocyclic ring from VQM intermediates, and introduced several different application examples in detail, all of which obtained excellent yields and enantioselectivities (Scheme 29) [86,87]. In both strategies, heteroatoms act as nucleophiles to attack the centre sp-C of VQM to complete the intramolecular closure. These reaction conditions are mild, can usually be completed in a short time, with high yields and enantioselectivities, and have broad application prospects in some functional natural products and drug synthesis.

**Scheme 30:**
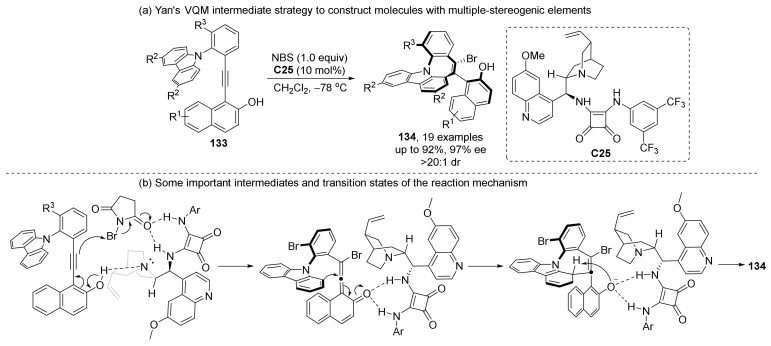
Construction of four chiral elements based on VQM intermediates.

Yan et al. developed a method for synthesizing four chiral elements in one step (**134**), based on their previous works (Scheme 30) [88]. The key step of this method is the generation of chiral VQM intermediates induced by H-bonding with squaramide catalyst. The resulting VQM intermediates not only act as an axially chiral element, but also hinder the free rotation of C-N bonds, resulting in the formation of a prochiral axis. A second chiral axis was selectively formed via steric hindrance of the squaramide catalyst.

**Scheme 31:**
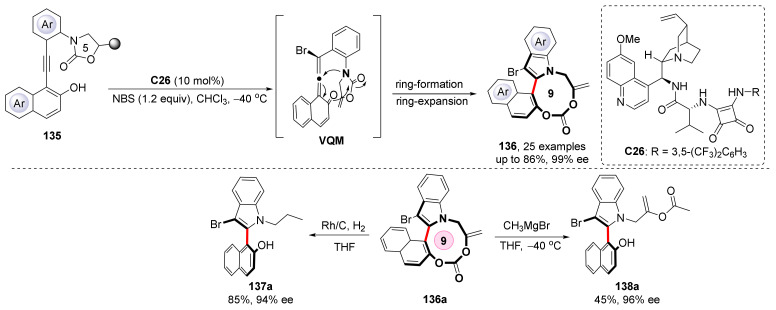
Synthesis axially chiral biaryls with nine-membered carbonate bridge.

The direct organocatalytic construction of nine-membered bridged biaryls with high stereoselectivity had not been achieved until 2022. Yan et al. revealed a strategy to obtain nine-membered carbonate-bridged biaryls based on their template reaction (Scheme 31) [89]. After the nucleophilic attack to the VQM intermediate, a series of electron transfers cause the naphthalene ring to restore aromatization; then, formed naphthol ions nucleophilically attack amide, in turn, to construct another ring. A subsequent ring-expansion reaction finally enlarges the ring into a nine-membered ring. The product can be transferred into other heterobiaryl compounds, exemplified in Scheme 31. Further research shows the products may have considerable inhibition effects on several cancer cell lines, which has considerable value and application prospects in the field of drug synthesis.

## 4. Proline Derivatives

**Scheme 32:**
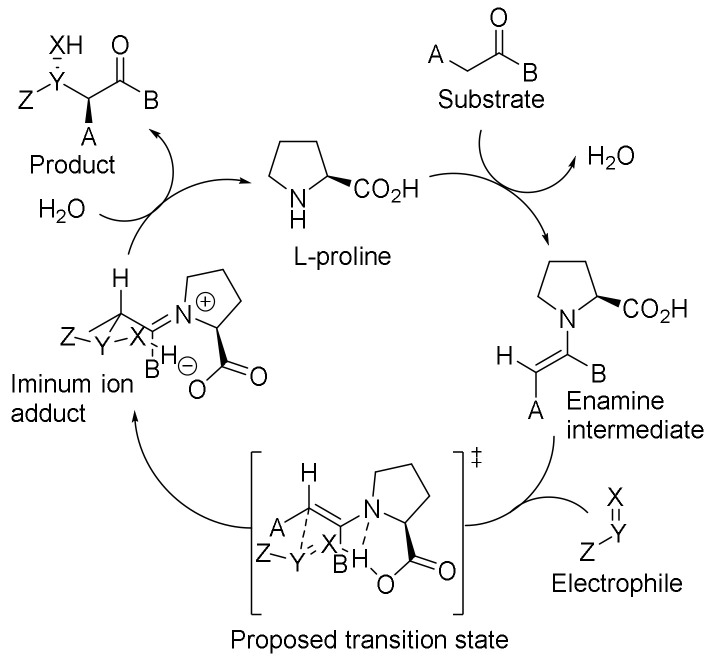
Reaction mechanism of *L*-proline as catalyst.

Examples of proline-catalyzed reactions have been reported since the 1970s, but did not receive much attention from researchers until the early 2000s. Proline was initially used as a metal ligand for organometallic catalysis, and its catalytic effect was not discovered until later. It is mainly used in the activation of carbonyl compound due to its nucleophilic property of the N atom in the ring [90,91]. By 2002, researchers had understood the mechanism of asymmetric catalysis and applied it to various asymmetric reactions (Scheme 32) [92]. To date, reactions using proline derivatives as catalysts are still emerging one after another, occupying an important position in the field of asymmetric synthesis where various catalysts blossom.

**Scheme 33:**
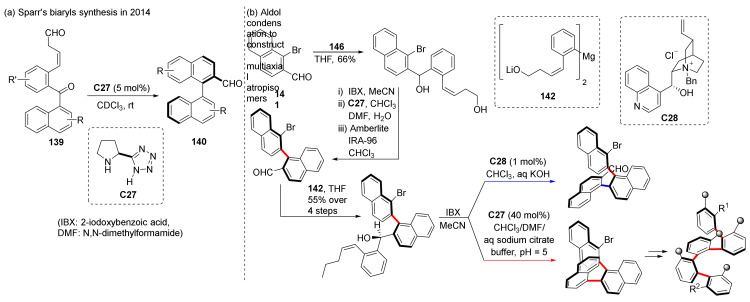
Sparr’s condensation strategy and synthesis of multiple stereogenic axes.

Sparr et al. used this catalyst in the construction of axially chiral compounds back in 2014. They used the designed substrate **139** to perform asymmetric aldehyde-ketone condensation catalyzed by *L*-proline derivative **C27**, and directly obtained the axially chiral biaromatic products **140** (Scheme 33) [93]. This strategy can efficiently transfer the stereochemical information of the catalyst into the axial chirality of tri-*ortho*-substituted biaryls [94]. Via functionalization of naphthalene rings and catalyst-controlled stereodivergent synthesis of helical oligo-1,2-naphthylenes, up to four stereogenic axes were successfully synthesized in 2018 [95,96]. Until then, this was the first example of a catalyst-controlled, stereodivergent synthesis of compounds with multiple stereogenic axes [97].

**Scheme 34:**
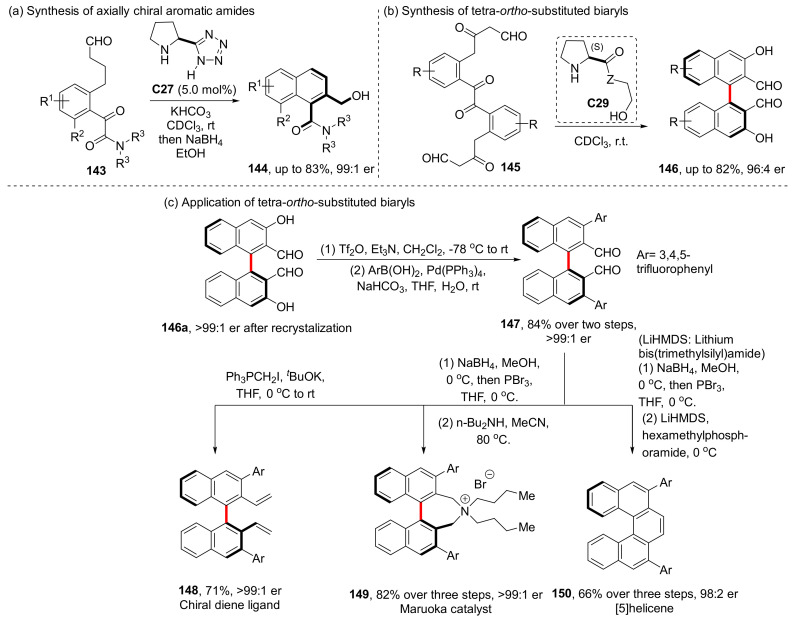
Extension of Sparr’s condensation strategy.

Additionally, they used the same catalyst and the same aldol condensation strategy to synthesize axially chiral aromatic amides and tetra-*ortho*-substituted biaryls, respectively, in 2016 and 2019 (Scheme 34) [98,99]. The highest yield was more than 80%, and the highest enantioselectivity was 99:1 er. Through two steps, the product **150a** can be converted into chiral diene ligand **152** with >99:1 er. It can also be transformed into Maruoka catalyst or [5] helicene in three steps (with >99:1 er and 98:2 er, respectively).

**Scheme 35:**
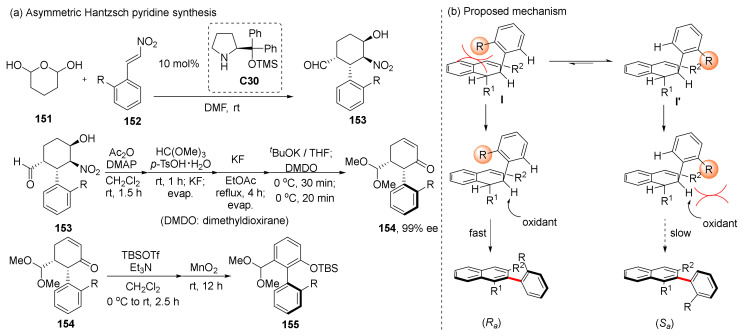
Tandem Michael/Henry reaction and oxidation mechanism.

Hayashi et al. reported in 2007 that four chiral centers were established in a tandem Michael/Henry reaction, in which diphenylprolinol silyl ether **C30** was the catalyst (Scheme 35) [100]. Based on this research, they attempted to construct chiral axes by oxidizing this product in 2019 [101], and obtained up to 98% ee of axially chiral product **155**. In the process of oxidation, they observed that the configuration of product was opposite to the stable conformation of the centrally chiral substrate, and they conducted in-depth research on this in 2020 [102]. Due to the steric hindrance of the R group, the intermediate **I’** in the dominant conformation has a slower oxidation rate due to steric effect with oxidant, which makes its oxidation products only a small part of the final product.

**Scheme 36:**
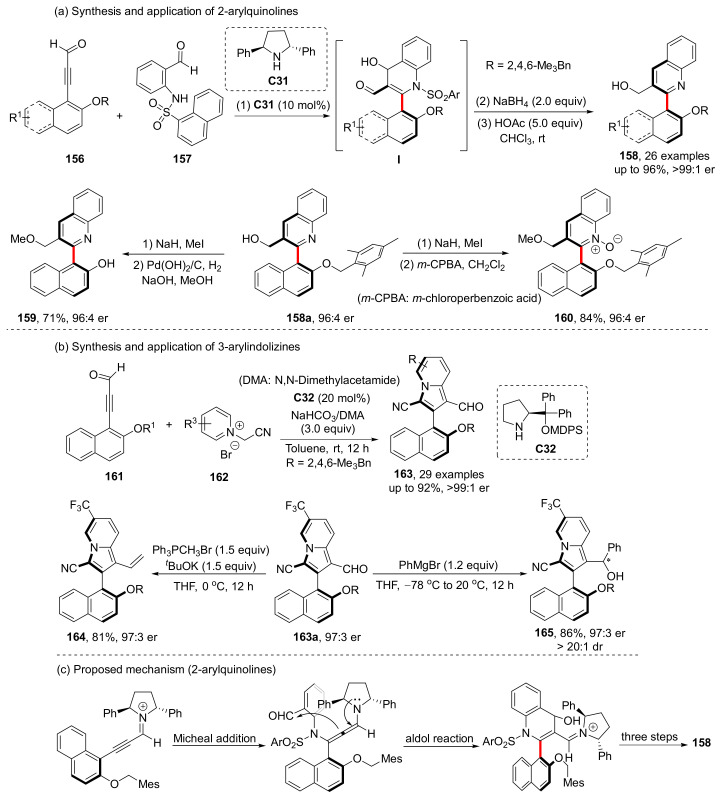
Chiral amine catalyzed synthesis of arylquinolines.

In 2021, Wang et al. synthesized the axially chiral 2-arylquinolines **158** and 3-arylindolizines **163** through an allene intermediate formed by chiral catalyst condensed with propiolaldehyde substrate **156** (Scheme 36) [103,104]. After the cyclization is completed, the chiral catalyst hydrolyzes away and the aldehyde group is restored. Both 2-arylquinolines and 3-arylindolizines have good yields and enantioselectivities (highest 96% yield, >99:1 er and 92% yield, >99:1 er, respectively). The former can be further converted to axially chiral QUINOX and isoquinoline analogues while maintaining enantioselectivity, and the synthetic transformations of the latter can also give excellent yields and enantioselectivities.

**Scheme 37:**
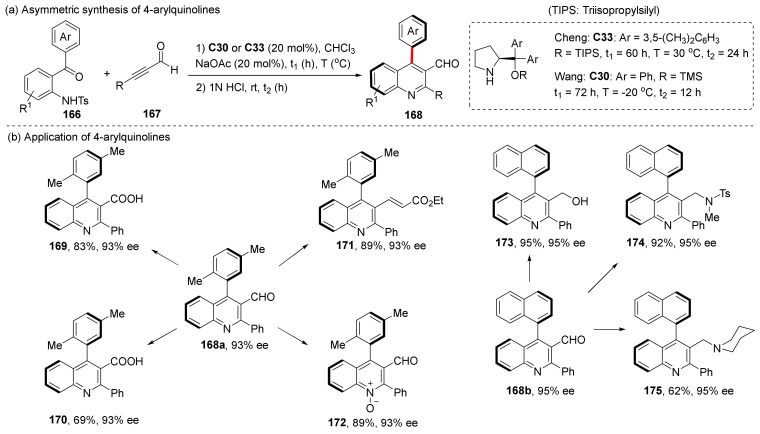
Cascade aza-Michael/aldol reaction and application.

In the same year, Wang et al. and Cheng et al. used chiral amines to catalyze the cascade aza–Michael/aldol reaction to synthesize 4-arylquinoline-3-carbaldehydes **168** (Scheme 37) [105,106]. The cyclization process can be referred to as the catalytic formation of allene intermediate via chiral amines described in the previous section. Since there are hydroxyl groups attached to the centrally chiral C, the chiral transition can be completed by adding acid and dehydration without introducing any oxidants. The products can be functionalized while maintaining high ee values, resulting in 4-arylquinolines containing a variety of functional groups (**169**–**175**).

## 5. *N*-Heterocyclic Carbenes (NHCs)

Since the first case of NHC-catalyzed enantioselective reaction was reported by Sheehan and Hunneman in 1966 [107], more and more chiral NHCs have been reported, including thiazolinylidenes, imidazolidinylidenes, imidazolinylidenes, and triazolinylidines [108,109]. Various chiral molecules with stereogenic centers can be prepared by chiral NHCs. Not surprisingly, NHC-catalyzed cycloaddition/cyclization to synthesize axially chiral molecules has also emerged. 

**Scheme 38:**
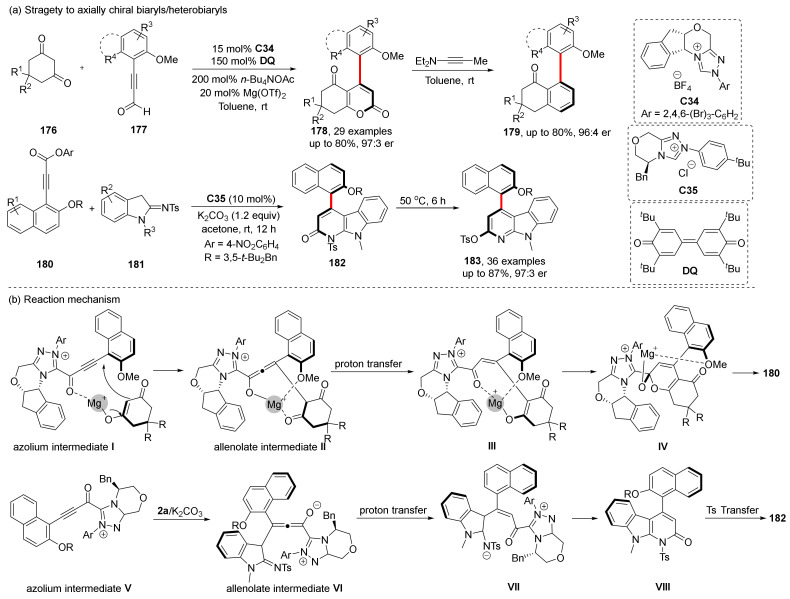
MHC-catalyzed cyclization via alkynyl acyl azolium.

Most axially chiral construction using NHC-catalyzed cycloaddition/cyclization concentrated on propiolaldehyde activation. In 2018, Wang et al. first reported such reactions for constructing axially chiral compounds. They designed alkynyl acyl azoliums as intermediates to synthesize α-pyrone-aryls, which can be further converted into axially chiral biaryls (Scheme 38) [110]. In this transformation process, the NHC catalyst combines with alkynaldehyde to produce azolium intermediate; then, it reacts with 1, 3-cyclohexadione to produce allenolate intermediate **II**, and finally undergoes proton transfer and cyclization to give product **179**. The axial chirality of product **179** stems from steric hindrance between naphthalene ring and 1,3-cyclohexadione induced by chiral NHC when the allenolate intermediates were formed. Subsequent processes do not change the chirality of the product; thus, the axial chirality of **179** is preserved. In 2021, Du et al. synthesized axially chiral 4-aryl-α-carbolines using a similar strategy, and up to 36 products were obtained with good-to-excellent yields and enantioselectivities (up to 87% yield, 97:3 er), which proved the wide applicability of the strategy (Scheme 38) [111].

**Scheme 39:**
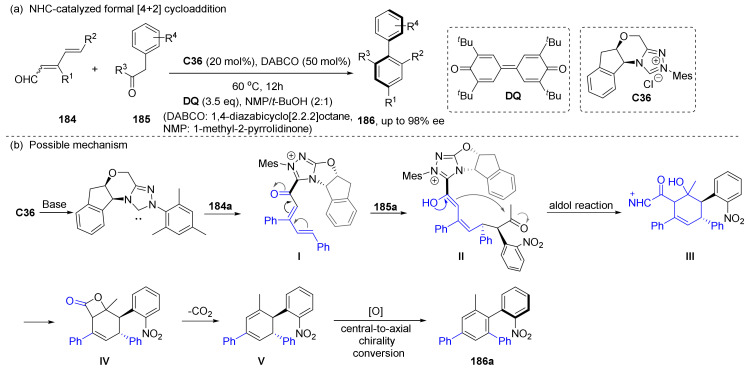
Asymmetric [4+2] cycloaddition reaction to synthesize axially chiral biaryls.

Zhu et al. reported an NHC-catalyzed asymmetric [4+2] cycloaddition reaction to synthesize axially chiral biaryls **186** in 2019 (Scheme 39) [112]. After two additions induced by chiral NHC, the centrally chiral intermediate **I** is constructed, and the central chiral product **V** then undergoes chiral transition to produce axially chiral product **186a** under the external oxidation conditions. The mechanism of chiral transition will be discussed in detail in the next section. Since the reaction requires a large amount of **DQ**, they also developed an electrochemical oxidation method, which also produced products with higher than 90% ee.

**Scheme 40:**
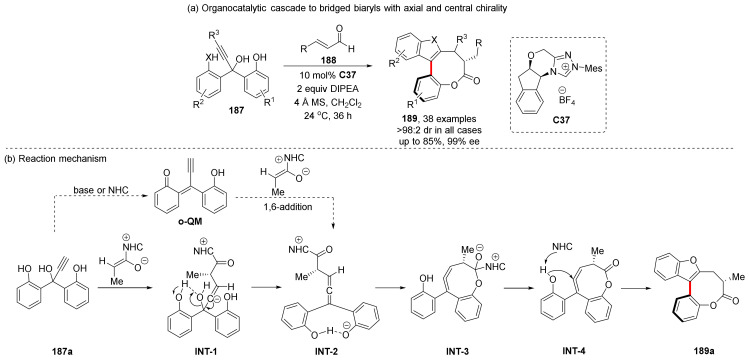
Axially chiral bridged biaryls synthesis and reaction mechanism.

In 2021, Zhao et al. designed another allene intermediate and directly used azolium as catalyst to successfully synthesize bridged biaryls **189** bearing axial and central chirality (Scheme 40) [113]. In the proposed mechanism, the alkynyl group is attacked by azolium formed by NHC and α-β-unsaturated aldehydes, and undergoes a series of electron transfers to dehydrate a molecule of water to form an allene-type intermediate (**INT-2**). The biaryl structure of this intermediate is not directly involved in the next reaction, but the NHC catalyst is removed to form an eight-member ring (**INT-4**); then, NHC catalyzes the phenol hydroxyl group to attack the biaryl intermediate to form the biaryl product. DFT calculations confirmed this process, and another reaction pathway via *o*-QM cannot occur due to the high-energy barrier.

**Scheme 41:**
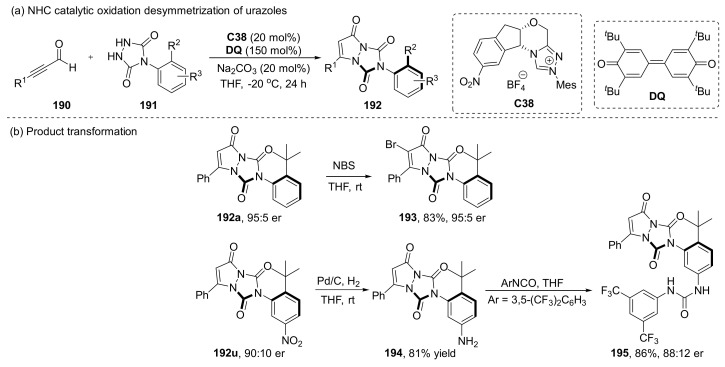
Chi‘s desymetrization strategy and its application.

Chi et al. combined urazoles with their characteristic NHC catalytic oxidation works to achieve the synthesis of axially chiral urazole derivatives **192** via NHC-catalyzed [3+2] cycloaddition desymetrization (Scheme 41) [114]. This reaction can be scaled up to gram-scale and retains 90% yield and 92:8 er, and the resulting products **192a** and **192u** can continue to functionalize while maintaining excellent chirality, which has great application potential in bioactive molecules and catalysis.

**Scheme 42:**
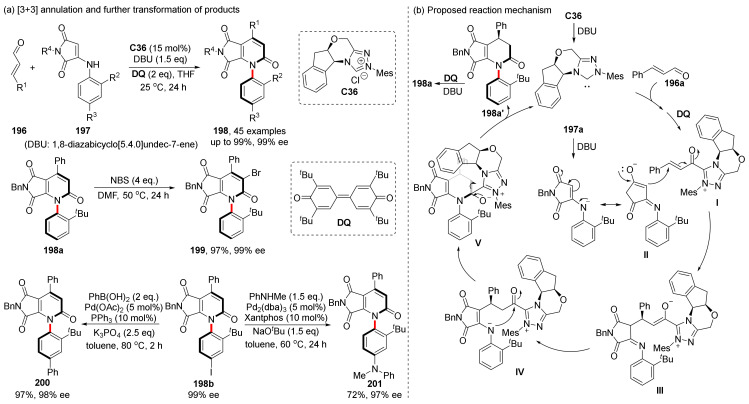
Hui‘s construction of C-N axis and mechanism.

Compared with C-C axially chiral compounds, C-N axially chiral compounds tend to racemize and lose chirality because one of the hybrid orbitals of N is occupied by electron pairs without forming a bond. In 2022, Hui et al. reported the atroposelective synthesis of pyrrolo[3,4-b]pyridines catalyzed by chiral NHC (Scheme 42) [115]. The aromatic amine derivative of maleimide and enal were used for [3+3] annulation, which showed excellent yields and enantioselectivities. In their proposed mechanism, the NHC catalyst first reacts with enal to form intermediate **I**, and Michael addition occurs with **197a** after base treatment. The obtained intermediate **III** undergoes proton transfer to form intermediate **IV**, and the nucleophilic addition of N to the carbonyl in the intermediate **IV** results in intermediate **V**. After the NHC leaves, compound **198a’** is oxidized by DQ to obtain the final product **198a**. The product **198a** can be further derived to produce **199** with 97% yield in the presence of NBS. Another product **198b** can be derived on the aromatic ring by transition-metal-catalyzed coupling reactions, yielding **200** and **201** with 97% and 72% yields, respectively. Compound **198a** is more stable than most C-N axially chiral compounds, with a 50.9 kcal/mol rotational energy barrier calculated via DFT.

**Scheme 43:**
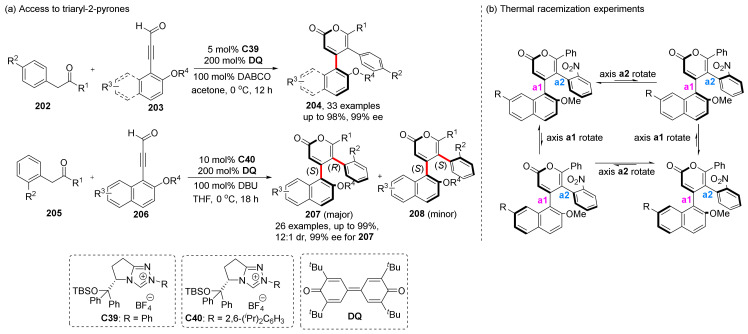
Lu’s muti-axially chiral triaryl-2-pyrones synthesis.

Lu et al. developed an NHC-catalyzed [3+3] annulation method to construct multi-axial chirality in 2023. Using 2-arylketones and ynals as substrates, they synthesized multi-axially chiral triaryl-2-pyrones (**204**, **207**) with up to 12:1 dr and 99% ee by constructing pyrone ring (Scheme 43) [116]. They conducted several thermal racemization experiments, and found that the product gradually transformed into diastereoisomers of other configurations above 80 °C. By analyzing the proportions of different configurations, it can be concluded that the rotational energy barrier of chiral axis **a2** is smaller than that of chiral axis **a1**, which is also confirmed via the DFT calculations.

## 6. Other Catalysts

In addition to the common organocatalysts discussed above, there are some other catalysts with excellent catalytic performance, which are also used in the construction of axially chiral compounds, which will be briefly described below.

**Scheme 44:**
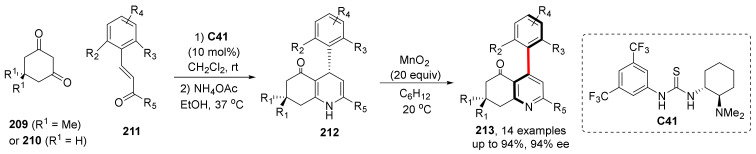
Hantzsch pyridine synthesis to construct chiral axis.

Thiourea derivatives and squaramide derivatives are commonly used as Brønsted acid catalysts, which have two N atoms that provide protons. Asymmetric cycloaddition/cyclization to construct axial chirality can be also catalyzed by introducing chiral groups to N atoms and controlling their steric hindrance. In 2016, Rodriguez et al. had already practiced this strategy with success (Scheme 44) [117]. They synthesized 4-arylpyridines by Hantzsch pyridine synthesis, using thiourea as the catalyst and MnO_2_ as the oxidant. This strategy firstly obtains intermediate 1,4-dihydropyridine **212** with central chirality, and then obtains the final product **213** via oxidation. Subsequently, in 2017 and 2020, they used this method to synthesize furan derivatives with one chiral axis and two chiral axes, and, respectively, obtained excellent results of up to 98% ee for the former and >20:1 dr, >99% ee for the latter [118,119].

**Scheme 45:**
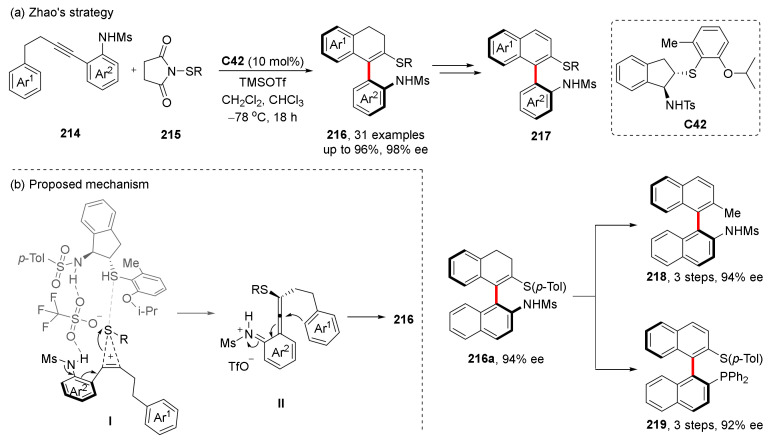
Carbothiolation of alkynes via aza-VQMs.

In 2019, Zhao et al. disclosed their progress on electrophilic carbothiolation of alkynes catalyzed by chiral sulfide **C42** (Scheme 45). Through this strategy, they successfully constructed axially chiral vinyl–aryl amino sulfides **216**, which can be easily converted into many valuable difunctionalized compounds (**217**–**219**) [120]. In this reaction, the chiral sulfur catalyst first binds to RS^+^ and then releases RS^+^; it reacts with the substrate alkyne to form a VQM-like structure through the formation and ring-opening of the sulfur-containing ternary ring (Scheme 45b). During the ring-opening process, OTf ion-mediated hydrogen bonding and the interaction between S and S control the configuration of the allene structure, thus determining the final chiral axis configuration. Then, the aromatic ring Ar^1^ attacks the central C atom of allene and rearomatizes to obtain product **216**.

## 7. Summary and Outlook

In this review, several organic catalysts commonly used in the construction of axial chirality via asymmetric cycloaddition/cyclization strategies are briefly introduced. The wide variety of intermediates and transition states of reaction experience symbolizes the breadth and depth of researchers’ exploration in this field. 

At present, an increasing number of chiral catalysts have been synthesized, and their mechanisms of catalytic activation have been further studied. With a better understanding of these kinds of reaction mechanisms, researchers have been able to design reactions and select substrates based on desired products. Despite certain achievements, there are still various challenges in this field of organocatalytic synthesis of axially chiral molecules that are likely to be addressed in future studies.

As shown above, great progress has been made in the synthesis of atropisomers through central-to-axial chirality conversion strategy. However, the current oxidation mechanism in the central-to-axial chirality conversion is not good at predicting the transformation results of the axially chiral configuration, which depend on the competing results of the rotational energy barriers and the corresponding oxidation rates of the two conformations. The theoretical research in this field remains to be explored and discovered. Moreover, as a more efficient and step economic strategy for the construction of axially chiral molecules, examples of organocatalytic cycloaddition or cyclization of alkynes are still less. The development of new activation models for activation of alkynes is highly desirable.

The in-depth study of catalyst and substrate interaction has led to more ideas of catalyst design and a wider selection of substrates. With further research in this field, more types of reaction mechanisms using new organocatalysts containing new functionalities (e.g., halogen bond dornors, frustrated Lewis pairs, covalent radical precursors) will be developed, giving future researchers higher-yield and higher-enantioselective reaction strategies to choose from. We hope that this review will provide reference and guidance for chemists who are new to organocatalytic cycloaddition/cyclization for the construction of axial chirality or who are interested in this field.

## Data Availability

Not applicable.
